# Saccadic suppression in schizophrenia

**DOI:** 10.1038/s41598-021-92531-2

**Published:** 2021-06-23

**Authors:** Rebekka Lencer, Inga Meyhöfer, Janina Triebsch, Karen Rolfes, Markus Lappe, Tamara Watson

**Affiliations:** 1 Institute of Translational Psychiatry, University of Münster, Albert-Schweitzer-Campus 1, Geb. A9a, 48149 Münster, Germany; 2 Otto-Creutzfeldt Center for Cognitive and Behavioral Neuroscience, University of Münster, 48149 Münster, Germany; 3 Department of Psychiatry and Psychotherapy, University of Lübeck, 23538 Lübeck, Germany; 4 Institute of Psychology, University of Münster, 48149 Münster, Germany; 5 School of Psychology, MARCS Institute for Brain, Behaviour and Development, Western Sydney University, Penrith, NSW 2751 Australia

**Keywords:** Neuroscience, Psychiatric disorders

## Abstract

About 40% of schizophrenia patients report discrete visual disturbances which could occur if saccadic suppression, the decrease of visual sensitivity around saccade onset, is impaired. Two mechanisms contribute to saccadic suppression: efference copy processing and backwards masking. Both are reportedly altered in schizophrenia. However, saccadic suppression has not been investigated in schizophrenia. 17 schizophrenia patients and 18 healthy controls performed a saccadic suppression task using a Gabor stimulus with individually adjusted contrast, which was presented within an interval 300 ms around saccade onset. Visual disturbance scores were higher in patients than controls, but saccadic suppression strength and time course were similar in both groups with lower saccadic suppression rates being similarly related to smaller saccade amplitudes. Saccade amplitudes in the saccadic suppression task were reduced in patients, in contrast to unaltered amplitudes during a saccade control task. Notably, smaller saccade amplitudes were related to higher visual disturbances scores in patients. Saccadic suppression performance was unrelated to symptom expression and antipsychotic medication. Unaltered saccadic suppression in patients suggests sufficiently intact efference copy processing and backward masking as required for this task. Instead, visual disturbances in patients may be related to restricted saccadic amplitudes arising from cognitive load while completing a task.

## Introduction

In daily life, we make about 20,000 saccades per day at speeds of up to 900°/s. Saccadic suppression describes the phenomenon of reduced visual sensitivity ± 50 ms around saccade onset, depending mainly on the contrast and spatial frequency of the visual environment^1^. Saccadic suppression thus prevents blurred, distorted visual perceptions caused by the high saccadic speed. It is suggested to rely on two mechanisms. The first mechanism comprises an efference copy (EC) of the oculomotor command sent to the visual system to proactively reduce the effective contrast of visual input around saccade onset^2–6^. Supporting this model, specific neuronal correlates of saccadic suppression have been demonstrated in core areas of the dorsal visual stream and have been linked to specific types of EC^7^. One EC type, which has been related to saccadic suppression, is thought to originate from transient burst neurons in the colliculus and codes for saccade onset^8,9^. Another EC type is suggested to consist of more slowly varying signals transferring a continuous read out of eye position to the parietal cortex^10–14^. Second, backward masking by the higher contrast scene available once the eye has stopped moving is thought to obliterate the peri-saccadic input from conscious perception^15–21^. Thus, while ECs are thought to represent preparatory mechanisms actively working to counter the perceptual effect of high saccadic eye velocity, masking is considered the result of temporal integration within the visual system. Both mechanisms appear to be potentially disturbed in schizophrenia and have been used to explain underlying mechanisms of psychotic symptoms.

For instance, disturbances in the use and quantification of EC based prediction errors have been used to explain delusions of alien control, which, according to this model, could result from the misinterpretation of own actions as being induced by external forces^22^. In this sense, the oculomotor system offers an ideal model linking psychotic experiences to basic physiological processes. To support the hypothesis of altered EC use in schizophrenia, different impairments of eye movement control in patients have been listed. These impairments comprise abnormal double-step saccades, in which the accurate second saccade requires accounting for the metrics of the first saccade coded by EC^23,24^, greater peri-saccadic mislocalization^10^, response inhibition during antisaccades and a greater reduction of smooth pursuit eye velocity in intervals with blanking of the visual target^24^. Reports about neural abnormalities within the visual dorsal stream during eye movements in patients, e.g. smooth pursuit eye movements^25,26^, are consistent with this hypothesis. Additionally, structural alterations within an EC pathway from the mediodorsal thalamus to frontal eye fields (FEF) have been reported in patients^27^. Conversely however, there are also several observations that do not support altered EC use in schizophrenia. These comprise reports of unimpaired peri-saccadic compression^10^, unaltered saccadic adaptation strength and unaltered adaptation-induced mislocalization^28^ in patients. Furthermore, an even stronger reliance on EC signaling in patients has been suggested from studies demonstrating slower eye velocity decrease after switching off a visual target during ongoing smooth pursuit^29^. In this situation, eye movements are solely driven by predictive mechanisms relying on EC processing. In line with this, during the blanking task greater FEF activation has been observed in patients compared to controls, probably reflecting enhanced recruitment of predictive meachnisms in FEF^30^. One explanation for these ambiguous findings on EC processing in schizophrenia is that there is not a general deficit of EC processing in schizophrenia but that since different eye movement tasks involve EC mechanisms in different circuitries, each may be differently disturbed. Further studies in schizophrenia are therefore needed to provide additional evidence to the pattern of impaired and unimpaired EC mechanisms involved in perception and eye movement control. In particular, it will be important to investigate possible links between these mechanisms and other perceptual disturbances reported by patients as discussed below.

In contrast to the active mechanism specific to the sensory outcome of motor actions discussed with EC signaling, backward masking is considered the result of the usual temporal functioning of the visual system. Numerous alterations of backwards masking in schizophrenia have been reported, with longer mask effects resulting in a longer attenuation of the percept of the pre-mask stimulus^31^. A generalized problem with enhancement of weak but functionally important signals^32^ or altered activity in the magnocelluar system are both discussed as possible mechanisms^33^.

Based on the ambiguous findings we have outlined and the visual disturbances experienced by up to 40% of people with schizophrenia or those prone to psychosis^34–36^, we designed a task to measure saccadic suppression in patients. We also assessed participants on the experience of discrete visual disturbances such as visual sensitivity, visual echoes, perceptual distortions and the like^36^. These disturbances are clinically highly relevant as they impact patient’s well-being^37^, are associated with treatment responses^38^, and with cognitive abilities^39^. The mechanisms underlying visual disturbances in schizophrenia are unclear^33,36,40,41^ and the relationship with EC processing has not been explored. We expected that if EC signaling or processing is impaired in patients, saccadic suppression should be weaker and therefore result in insufficiently reduced visual sensitivity around saccade onset. This may provoke visual disturbances such as blurred visual perception or the perception of pseudomovements elicited by the high saccadic speed. Increased backward masking, on the other hand, should result in earlier and longer saccadic suppression around saccade onset which may also provoke phenomena of altered visual perception. Both aspects, reduced saccadic suppression strength as indicated by smaller suppression index as well as earlier and longer saccadic suppression, can be studied by relating saccade onset to the perception of a probe flashed while a saccade is in flight.

## Materials and methods

### Participants

Seventeen patients from in- and outpatient services of the Department of Psychiatry and Psychotherapy at the University of Münster met DSM V-criteria for a schizophrenia spectrum disorder including schizophrenia (N = 10) and schizoaffective disorders (N = 7)^42^. Diagnoses were determined at consensus conferences using all available clinical data. Eighteen healthy control participants, all without a history of Axis I disorders according to the Structured Clinical Interview for DSM IV^43^ or any known history of psychotic disorder in first-degree relatives were also recruited by newspaper advertisement and postings on noticeboards. Patients and healthy participants did not differ on either age, gender distribution or years of education, Table 1. In addition to standard clinical characterization including the Positive and Negative Syndrome Scale^44^, all participants were assessed for the experience of visual disturbances within the last three month using the respective section of the Schizophrenia Proneness Instrument, Adult version (SPI-A)^45^. In this section of the SPI-A severity and frequency, ranging from never (score = 0) to daily (score = 6), of 14 visual phenomena were rated including (1) changed perception of body or face of others, (2) hypersensitivity to light/optic stimuli, (3) photopsia, (4) micropsia, macropsia, (5) near and tele-vision, (6) metamorphopsia, (7) changes in colour vision, (8) changed perception of patient’s own face, (9) pseudomovements of optic stimuli, (10) diplopia, oblique vision, (11) disturbances of the estimation of distances or sizes, (12) disturbances of the perception of straight lines/contours, (13) maintenance of optic stimuli, “visual echoes”, and (14) partial seeing including tubular vision. Individual sum scores over all items reflecting the burden by visual disturbances were calculated after principal component analysis had identified one major factor explaining 31.5% of variance (Eigenvalue = 4.7).

**Table 1:** Sociodemographic and clinical characteristics of patients with schizophrenia (N = 17) and healthy controls (N = 18) of similar age participating in a saccadic suppression task. ^a^Positive and negative syndrome scale^44^. ^b^Schizophrenia proneness instrument, adult version^45^. ^c^According to Ref.^46^.

	Schizophrenia patients N = 17	Healthy controls N = 18	Group comparison
Age, mean (SD), years	35.7 (10.1)	34.2 (11.6)	T_(33)_ 0.402, p = 0.690
Sex (% male)	14 (82%)	14 (78%)	Χ^2^ = 0.144, p = 0.735
Years of education, mean (SD)	15.2 (2.1)	15.8 (1.7)	T_(33)_ 1.018, p = 0.316
Illness duration, mean (SD), years	10.6 (7.6)	n. a	n. a
PANSS^a^ positive, mean (SD)	16.29 (5.90)	7.11 (0.47)	T_(33)_ 6.584, p < 0.001
PANSS^a^ negative, mean (SD)	17.00 (7.24)	7.06 (0.24)	T_(33)_ 5.832, p < 0.001
PANSS^a^ general, mean (SD)	34.00 (8.80)	16.22 (0.43)	T_(33)_ 8.571, p < 0.001
PANSS^a^ total, mean (SD)	67.29 (14.20)	30.39 (0.85)	T_(33)_ 11.017, p < 0.001
Visual perception disturbances, mean SPI-A^b^ sum score (SD)	4.65 (6.47)	0.28 (0.83)	T_(33)_ 2.843, p = 0.008
**Medication status**			
Chlorpromazine equivalents^c^, median	821 mg	0	n.a
Antidepressants, N (%)	5 (29%)	0	n.a
Mood stabilizer, N (%)	2 (12%)	0	n.a

Exclusion criteria for all participants included: (1) any known systemic or neurological disease or history of head trauma with loss of consciousness > 10 min, (2) any ophthalmological disease, (3) regular intake of any tranquilizers, i.e. benzodiazepines, (4) no substance dependence for at least 1 year and no substance abuse within the last month according to DSM V criteria, (5) corrected or uncorrected decimal visual acuity of less than 0.8 tested by Landolt rings, and (6) no evidence for red-green-deficiency as assessed by the Ishihara test. At the time of testing, patients were on stable antipsychotic medication for at least two weeks. All individual antipsychotic doses were converted into daily chlorpromazine equivalents^46^, Table 1. All participants gave written informed consent according to the Declaration of Helsinki. The study was approved by the ethics committee of the University of Münster.

### Experimental design

All eye movement recordings were conducted in the same room at the Department of Psychiatry and Psychotherapy under constant conditions regarding illumination (0.01 cd/m^2^), noise and standardized task instructions. Subjects were seated 70 cm in front of a CRT monitor (Samsung SyncMaster 950p Plus, diagonal of 45.5 cm (18″), resolution of 1280 × 1024 pixels, refresh rate 85 Hz) with their heads stabilized by a chin rest with forehead restraint. Care was taken to avoid any visual stimulation other than that displayed on the monitor to prevent the use of landmarks or reference for localization. For eye movement recordings the desktop mount EyeLink 1000 system (SR Research Ltd., Mississauga, Ontario, Canada) was used. Data were recorded at 500 Hz binocularly and recordings of the eye providing the best data quality were used for analysis. This was checked systematically for all recordings. Stimuli were generated by custom made programs using Psychtoolbox (Version 3.10, Brainard 1997) for Matlab R2016a (The MathWorks, Natick, MA, USA). The whole testing procedure took about 40 min. Each saccade task was preceded by a nine-point eye tracker calibration allowing for a deviation of 0.25° and a one-point drift correction.

#### Saccadic suppression task

**Figure 1:**
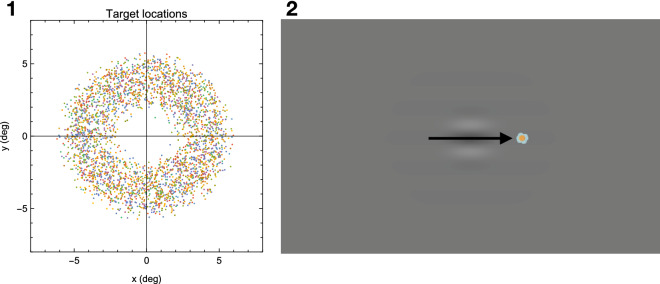
(1-**1**) Summary of target locations across all trials and participants (indicated by different colors). The distance between two successive target locations was always 7°. (1-**2**) Example of a saccadic suppression trial with a probe displayed by a Gabor appearing halfway (3.5°) along the saccade trajectory (depicted as arrow). Gabor grating was oriented parallel to saccade trajectory.

Targets were designed as little flowers (30 pixels, 0.7°) each consisting of a colored circle (10 pixels) surrounded by six equally colored circles (each 10 pixels), i.e. petals, of different color than the middle circle. The color of the middle circle was selected randomly from the full RGB spectrum, while the color of the respective petals was selected at random from a smaller range of the RGB spectrum offset from the value of the middle circle. Flower targets were successively displayed for 2000 ms each on a grey background (23–27 cd/m^2^) at random positions, each being apart from the next by 7° (Fig. 1-**1**). Presentation sequences of flower target positions were matched between the two groups with one participant from each group following an identical sequence. Subjects were instructed to make a saccade towards each flower as soon as it appeared. Around saccade onset, a Gabor stimulus (phase = 90°, spatial frequency = 0.43 cyc/°, half-width = 1.2°), oriented parallel to the saccade trajectory and centered halfway (3.5°) to the target/flower was displayed for one frame (11 ms; Fig. 1-**1**). Distribution of Gabor flashes relative to saccade onset was similar in both groups (Fig. 1-**1**). Subjects were asked to press a button anytime they noticed the Gabor using the space key on the keyboard.

Because the beam of the CRT monitor draws the stimulus in a sequential fashion each frame and the saccades sweep across the monitor at high velocity the stimulus presented on the retina will be slightly distorted from the stimulus presented on the screen, particularly during up- and downward saccades. Since the speed of the monitor beam is about 2000°/s and the speed of the saccade on the order of 200°/s this would produce an elongation or compression of the retinal stimulus by at most 10%. While such a distortion might slightly affect differences in detection rates between upward and downward saccades it should not produce any difference between groups, also because presentation sequences of target positions were matched between the two groups.

The experiment started with 40 preparatory trials with the first 12 trials serving as training trials to familiarize participants to the task. Here, the probe, i.e. Gabor stimulus, was either presented 50 ms (k = 4 trials) or 400 ms (k = 4 trials) after presentation of the flower target, or no probe was presented (k = 4 catch trials). On the trials with a probe, the Gabor always had a high contrast to provide practice. These trial types were randomly presented.

During the following 28 trials individual stimulus contrast thresholds for a 90% detection rate outside the time window of saccadic suppression were defined in each participant using a Bayesian QUEST-algorithm^47^. In 24 of those trials, the probe was presented 400 ms after presentation of the flower target. In 4 trials that were randomly interspersed the probe was shown 50 ms after presentation of the flower.

The resulting, individually established Gabor contrast was used during the following 200 test trials divided into 10 blocks. Here, the aim was to present the probe before, on and after saccade onset covering a range of about 300 ms around saccade onset, thus a probe was always presented. During the testing procedure latency was recalculated online across the 20 trials of each block to adjust the timing of the probe presentation accordingly. Only saccades with latencies ≥ 80 ms and amplitudes ≥ 3.5° were considered here.

#### Data analyses of the saccadic suppression task

We used the standard SR Research-Algorithm (”Eye Tracking with the EyeLink Eye Trackers—SR Research Ltd.”, 2019) to detect saccades (> 30°/s, > 8000°/s^2^, latency > 80 ms) and define their characteristics. Baseline probe detection rate was defined in an interval 200 ms to 70 ms before saccade onset while peri-saccadic detection rate was defined as the running weighted mean detection rate at saccade onset smoothed with a Gaussian filter (δ = 10 ms). Parameters of interest included: (1) baseline detection rate, (2) suppression index ((baseline detection rate − peri-saccadic detection rate)/baseline detection rate), (3) saccade amplitude, (4) its standard deviation reflecting amplitude variability, (5) saccadic curvature, since saccades, particularly in oblique direction, are not entirely straight, (6) absolute saccade error, (7) absolute distance to probe location, (8) saccade latency and (9) its standard deviation reflecting latency variability, Table 2.

**Table 2:** Saccade metrics of patients with schizophrenia (N = 17) and healthy controls (N = 18) derived from a saccadic suppression task and an overlap saccade control task. Statistically significant group differences are indicated in bold.

	Schizophrenia patients N = 17	Healthy controls N = 18	Group comparison
**Saccadic suppression task, mean (SD)**			
Baseline detection rate in the interval 200 ms to 70 ms before saccade onset	0.95 (0.06)	0.98 (0.02)	**T**_**(33)**_** 2.386, p = 0.023**
Suppression index at saccade onset	0.74 (0.18)	0.78 (0.14)	T_(33)_ 0.811, p = 0.423
Amplitude (°)	4.61 (1.25)	5.61 (0.87)	**T**_**(33)**_** 2.774, p = 0.009**
Amplitude variability (°)	1.58 (0.26)	1.43 (0.32)	T_(33)_ 1.511, p = 0.140
Amplitude, 1st half (°)	4.70 (1.23)	5.74 (0.90)	***Time*: F**_**(1,33)**_** 14.084, p = 0.001** ***Group*: F**_**(1,33)**_** 7.630, p = 0.009** *Time x group*: F_(1,33)_ 0.547, p = 0.465
Amplitude, 2nd half (°)	4.51 (1.24)	5.47 (0.90)	
Saccadic curvature	0.16 (0.07)	0.12 (0.05)	T_(33)_ 1.910, p = 0.065
Absolute saccade error (°)	2.09 (0.56)	1.65 (0.38)	**T**_**(33)**_** 2.745, p = 0.010**
Absolute distance to Gabor location at saccadic endpoint (°)	2.62 (0.32)	2.7 (0.36)	T_(33)_ 0.634, p = 0.537
Latency (ms)	168 (46)	174 (25)	T_(33)_ 0.460 p = 0.649
Latency variability (ms)	128 (57)	101 (71)	T_(33)_ 1.225 p = 0.229
**Overlap saccade task, mean (SD)**			
Mean amplitude gain	0.9 (0.06)	0.93 (0.04)	T_(32)_ 2.001 p = 0.054
Mean latency (ms)	222 (55)	235 (41)	T_(33)_ 0.751 p = 0.458

#### Overlap saccade task

In a separate session, participants were additionally tested on standard visually guided saccades of 8° and 12° to left and right of a central fixation point (0.75°, 52.5 cd/m^2^) presented in randomized order (total of 32 trials). Each trial started with a central fixation point visible for 1500 to 2500 ms. Two-hundred ms before the central fixation point was extinguished the eccentric targets appeared and remained visible for 1500 ms (overlap-design). Subjects were instructed to make a saccade to the eccentric target as soon as it appeared on the screen. After the eccentric target was extinguished, the central fixation point appeared again to start the next trial.

#### Data analysis of the overlap saccade task

Eye movement recordings were calibrated, saccades and blinks were automatically detected and manually checked with an interactive program written in Matlab R2016a (The MathWorks, Natick, MA, USA). Parameters of interest were (1) latency of the first saccade (minimum latency > 80 ms), and (2) its amplitude to calculate gain (saccade amplitude/target amplitude > 0.5).

#### Statistical analyses

To compare group differences, we used independent sample t-tests, two-way mixed Analyses of Variance (ANOVA) and Chi- square tests whenever appropriate as implemented in the software package IBM SPSS version 25 (IBM Deutschland GmbH, Ehningen, Germany). Possible effects of clinical variables and medication status on saccadic parameters were evaluated by Pearson correlations and Spearman’s rank correlation, respectively.

## Results

### Saccadic suppression

**Figure 2:**
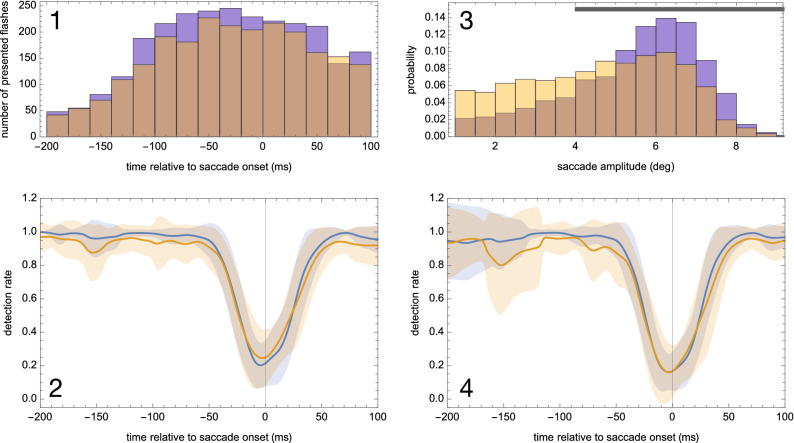
(**2-1**) Distribution of Gabor flashes relative to saccade onset in patients with schizophrenia (yellow) and healthy controls (blue). Overlap between groups is indicated in brown. Note, distribution of Gabor flashes was similar in both groups. (**2-2**) Mean probe detection rates with standard deviations related to saccade onset in patients with schizophrenia (N = 17) and healthy controls (N = 18). Detection rates are depicted as running averages. Detection rate around saccade onset did not differ between patients and controls, nor was there any evidence for prolonged backwards masking starting earlier in patients. However, baseline detection rate in the interval 200 ms to 70 ms before saccade onset was slightly, but significantly, lower in patients than controls. (**2-3**) Distribution of saccade amplitudes in patients with schizophrenia (yellow) and healthy controls (blue) during the saccadic suppression task. Overlap between groups is indicated in brown. Black bar indicates range of amplitudes used in the analysis below (**2-4**). (**2-4**) Similar to (**2-2**) but based on only amplitudes > 4°. Note, the suppression at saccade onset is even more similar between the groups than in (**2-2**).

Figure 2-2 shows running averages of the detection rate over time relative to saccade onset. In both groups, baseline detection rate between 200 and 70 ms before saccade onset was slightly higher than expected from the QUEST procedure. This might reflect a certain training effect in both groups during the experiment. It could also have resulted from a few trials in the QUEST procedure in which an inadvertent saccade occurred within 50 ms of the presentation time of the Gabor stimulus, thus producing some suppression. Post-hoc analysis indicated that this happened similarly in both groups in a small number of trials (mean controls = 3.50, SD = 2.75; mean patients = 4.82, SD = 2.63; T = 1.45, p = 0.16). These trials would have affected the threshold estimate such that the baseline in the subsequent experiment trials would be slightly higher.

Baseline detection rate was slightly lower in patients (mean 0.95, standard deviation (SD) 0.06) than controls (mean 0.98, SD 0.02; T_(33)_ 2.386, p = 0.023). As expected from previous reports, saccadic suppression occurred within about ± 50 ms around saccade onset. This was true in both groups. The saccadic suppression index did not differ between groups (patients: mean 0.74, SD 0.18; controls: mean 0.78, SD 0.14; T_(33)_ 0.811, p = 0.423) indicating unimpaired saccadic suppression in patients with schizophrenia. There was also no evidence that saccadic suppression started earlier or lasted longer in patients than controls. Follow-up Bayesian analysis with the software package JASP (JASP Team (2020). JASP Version 0.12.2) provided Bayes factors BF_01_ between 2.38 and 4.0, depending on prior width, and hence favored equal saccadic suppression between the groups with moderate evidence.

Saccade latency and saccadic curvature did not differ between the groups either, Table 2.

**Figure 3:**
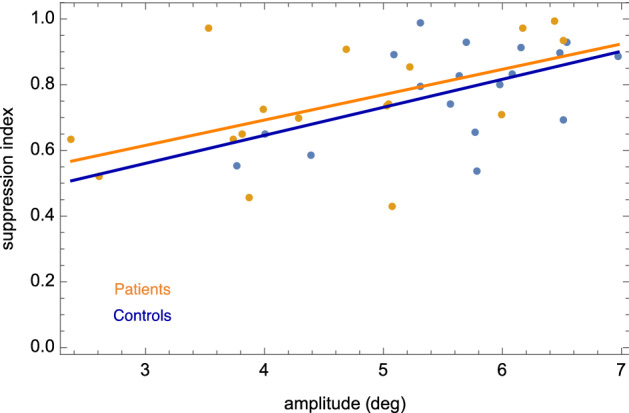
In both groups, smaller saccade amplitudes were related to a smaller suppression index.

Despite the finding of unimpaired saccadic suppression in patients, patients made considerably smaller saccades (patients: mean 4.61°, SD 1.25; controls: mean 5.61°, SD 0.87; T_(33)_ 2.774, p = 0.009, Fig. 2-3) resulting in a significantly larger saccade error (patients: mean 2.09°, SD 0.56; controls: mean 1.65°, SD 0.0.38; T_(33)_ 2.745, p = 0.010). Note that amplitude variability was similar in both groups, Table 2. Testing the possibility that patients, in contrast to controls, may have directed their saccades rather towards the expected Gabor location than towards the flower location we found that the absolute distance to the Gabor location from saccade landing location was similar in both groups, Table 2. A higher suppression index was also related to larger saccades (patients: r =  − 0.539, p = 0.026; controls: r =  − 0.528, p = 0.024; Fig. 3) and lower saccade latency variability (patients: r = − 0.519, p = 0.033; controls: r =  − 0.601, p = 0.008) in both groups.

In follow-up analyses we further tested the possibility that smaller saccade amplitudes in patients may have confounded the direct comparison of saccadic suppression indices between groups as the magnitude of saccadic suppression varies systematically with saccade amplitudes^48^. Rerunning analyses including only saccades > 4° revealed suppression indices at saccade onset of 0.825 (± 0.170) for patients and 0.828 (± 0.122) for the controls (n.s. T = 0.055, p = 0.956). The baseline detection rates remained as before (0.95 ± 0.07 for patients and 0.98 ± 0.03 for controls), Fig. 2-4.

### Relation of saccadic suppression to visual disturbances and other clinical variables

As expected, patients indicated higher mean burden by visual disturbances (referring to last three month) than controls (patients: mean sum score 4.65, SD 6.47; controls: mean sum score 0.28, SD 0.83; T_(33)_ 2.843, p = 0.008). Higher sum scores were related to smaller saccade amplitudes in patients (r = -0.526, p = 0.015, R^2^_adjusted_ = 0.228) with saccade amplitudes explaining 23% of variance in sum scores. This association was absent in controls (r = 0.055, p = 0.414, R^2^_adjusted_ =  − 0.059). Since the summed visual disturbance scores were not normally distributed we confirmed these results with a Spearman rank-order correlation which resulted in basically the same findings, e.g. the correlation of visual disturbances with saccade amplitudes in patients (Rho = 0.611, p = 0.009), which was absent in controls (Rho = 0.038, p = 0.882). No other saccadic parameter, including the saccade suppression index, was related to the sum score of visual disturbances in patients. There were also no significant correlations of saccadic parameters with symptom ratings on the PANSS subscales or with Chlorpromazine equivalents.

### Overlap saccade task

In contrast to the finding of reduced saccadic amplitudes in patients in the saccadic suppression task, saccadic amplitudes and gains of overlap saccades were not reduced in patients, Table 2. Also, saccadic latencies did not differ between groups. As expected, saccadic latencies in the overlap saccade task were generally longer than in the saccadic suppression task.

## Discussion

In this study, we included a representative group of psychopathologically stable schizophrenia patients with predominately negative symptom expression all being able to perform the experiments. As expected, patients reported experiencing visual disturbances more frequently and more severely than control participants^36^. We were interested whether these visual perceptual distortions are related to altered saccadic suppression, i.e. a weaker or a longer reduction of visual sensitivity around saccade onset. Two mechanisms underlying saccadic suppression are suggested to be impaired in schizophrenia as was postulated from other oculomotor and visual perception tasks^31^. The first mechanism involves the active suppression of visual sensitivity around saccade onset resulting from processing of an EC of the saccade motor command coding for saccade timing and the spatial landing location from the superior colliculi^49^. Our main finding with respect to this mechanism shows that the strength and time course of saccadic suppression is unaltered in patients. In addition, by comparing saccade curvatures between groups we were able to account for the fact that saccades in the suppression task were partly oblique and saccade trajectories between groups may have resulted in different stimulus visibility. In particular, this analysis did not uncover a possible interruption of saccade trajectories in patients, which could have resulted from sub-threshold saccadic suppression impairments (see Table 2). Furthermore, saccadic suppression indices were unrelated to experiences of visual disturbances in patients. Together, our finding of unimpaired saccadic suppression strength and time course in patients does not support the hypothesis of impaired EC processing and cannot explain the phenomenon of visual disturbances in schizophrenia.

The second mechanism underlying saccadic suppression is visual backward masking provoked by high eye velocity during a saccade ‘greying out’ the structure in a scene which is then masked by the higher contrast of new visual input once the eyes have stopped moving^15^. As backwards masking has been described to start earlier in patients and be prolonged up to 250 ms^31^ we might have expected saccadic suppression effects to begin earlier in patients, which was not the case, see Fig. 2.

The third important finding from this study is that in both groups lower saccadic suppression indices were related to smaller saccade amplitudes. This appears to be a general phenomenon and is not specific to schizophrenia. In patients, smaller saccade amplitudes, in contrast to saccadic suppression indices, were indeed associated with higher frequency and burden by the experience of visual disturbances. Note, patients were found to make smaller saccades than controls during the saccadic suppression task, but not during the overlap saccade task. This constellation of findings implies that independently from unimpaired strength and time course of saccadic suppression, visual disturbances in schizophrenia are suggested to be associated with impaired generation of saccadic amplitudes depending on specific task demands.

Our finding of unimpaired saccadic suppression in patients makes an important contribution to the discussion about disturbed EC processing underlying psychotic symptom development^22,23^. Based on the model that EC signaling is ubiquitously present in sensorimotor systems but that several different EC type signals have been revealed in monkey^11^ and human studies^50^, unimpaired saccadic suppression supports the notion that EC processing in schizophrenia is not generally impaired. It may be the case however that EC processing is specifically impaired in patients depending on task requirements. Our finding is in line with a previous report about unimpaired saccadic compression in schizophrenia suggesting that EC signaling from the superior colliculi via the thalamus to the FEF is sufficiently intact in patients^10^. Notably, while saccadic compression was found to be unimpaired in that study, overall peri-saccadic mislocalization (also requiring EC signaling) was larger in the same patients relative to controls^10^. The authors concluded that, while one EC pathway seemed unaffected in patients, the EC from superior colliculi sent to the parietal cortex involved in the localization task seemed overdamped in schizophrenia, probably due to increased noise in the signal^10^. This led to the model that EC deficits in schizophrenia are specific to tasks that require continuous monitoring of ongoing EC signaling^10^. The unimpaired saccadic suppression reported here indicates sufficiently intact EC processing- and is consistent with our previous research showing slower but equivalent saccadic outward adaptation strength in patients with schizophrenia^28^. A finding also implying functional EC signaling from the cerebellum to FEF in patients.

Besides unimpaired saccadic suppression we also found no evidence for altered backwards masking from our task. Here the flower target would mask out the percept of the Gabor probe. One explanation for this negative finding is the fact that backward masking heavily depends on the duration of the presentation of the first stimulus and the time when the mask is presented, i.e. with stimulus onset asynchrony (SOA) of 0–100 ms. Unaltered saccadic suppression performance in patients in the interval 0–150 ms prior to target onset in the present findings suggests that backwards masking was of rather minor importance in our experiment.

Notably, we did find that patients made smaller saccades than controls in the saccadic suppression task. We found smaller saccades were related not only to lower saccadic suppression rates (in both groups, see Fig. 3), but were also related to the reported frequency and severity of visual disturbances in daily life in patients, explaining 23% of variance in visual disturbances. At the same time, unaltered saccades in the overlap saccade task imply basically intact oculomotor function in patients. Smaller amplitudes in patients during the saccadic suppression task could not be explained by the possibility that patients more often generated a saccade towards the expected Gabor position instead of following the explicit task instruction to make a saccade as quickly as possible towards the flower. Both groups showed similar absolute distances between the probe location and the saccadic endpoint as well as unaltered saccade latency and variability. This confirms the observation that patients were well able to follow task instructions, Table 2. One unexplored difference may be due to the suppression task requiring saccades in 2D space while overlap saccades were performed only in the horizontal plane. Another aspect is that compared to the single-task nature of the overlap saccade task our saccadic suppression task had a more dual-task nature, combining a perception task requiring a button press with a reflexive saccade task. Together, this leads to a model of saccadic amplitudes in patients being highly dependent on task requirements. We anticipate cognitive load (including visual processing load) is an important factor, with greater cognitive challenges resulting in smaller saccade amplitudes in patients, as observed here with the cognitively more challenging saccadic suppression task compared to the overlap task. This model is supported by previous studies, including our own work showing reduced saccadic amplitudes of self-generated exploratory saccades in patients when scanning static pictures or while watching movies including the aspect of cognitive load of a task^51–55^. We can only speculate about the causality of the relationship between small saccadic amplitudes in patients as seen with the saccadic suppression task and other visual exploration tasks on the one hand and considerable burden by visual disturbances in daily life reported by patients on the other hand. First, restricted free visual exploration behavior due to small saccades may result in less visual information sampling and misinterpretation of features in the visual environment provoking visual distrubances^51–55^. Second, due to visual perceptual disturbances patients may have difficulties in defining saccade landing positions and thus generate smaller saccades which may reflect more instable visual error processing as observed during saccadic adaptation^28^. However, unaltered individual amplitude variability in patients compared to controls makes this second explanation unlikely.

Beside aspects discussed above, there may be further limitations from this neurophysiological study in a clinical population. First, all patients were on stable medication excluding sedatives. Although daily chlorpromazine dose must be considered as rather high in our patient sample, we did not find any evidence for medication effects on saccade or suppression measures. Similarly, no associations were found between neurophysiological measures with any other clinical measures including illness duration, symptom expression on the PANSS or experience of visual disturbances. Second, sample size is an issue. Based on the current data, if any difference in saccadic suppression were present in patients, at best a larger sample size might uncover a very small reduction in saccadic suppression in patients. This would be a very minor effect relative to the large peri-saccadic reduction in effective stimulus contrast experienced by patients and we would argue that its functional significance would be in doubt. Third, our estimate of baseline contrast for a detection rate of 90% was done with only 24 trials of a QUEST procedure. More trials might have avoided differences in baseline detection rates revealed during the experiment. However, increasing the number of trials would have likely provoked effects of exhaustion, especially in patients, which we wanted to avoid. Note, that our measure of saccadic suppression, the suppression index, normalizes for baseline differences.

The main message from our findings suggests that basic EC processing as well as backwards masking mechanisms as needed for saccadic suppression are sufficiently intact in patients with schizophrenia and are unrelated to the experience of visual disturbances. In the light of the extensive debate on the role of EC processing in psychotic symptom development this adds to the complex pattern of altered but also unaltered mechanisms. Instead, the burden by visual disturbances in patients seems rather related to restricted saccadic exploration behavior arising from cognitive load of a task or spatial variability of saccades with patients being impaired in flexibly adapting saccadic behavior to cognitive challenges of a task.
